# Clinical Notes on Dysemia

**Published:** 1903-08

**Authors:** Louis J. Gravel

**Affiliations:** Physician-in-chief to the Hotel Dieu Hospital and chief of the laboratory, Montreal, Canada


### Clinical Notes on Dysemia.[1]

By LOUIS J. GRAVEL, M. D.,

Physician-in-chief to the Hotel Dieu Hospital and chief of the laboratory, Montreal, Canada.

THE word anemia is a misnomer and serves to perpetuate an erroneous idea of the conditions to which it is applied. It had its origin at the time when scientific medicine was in its infancy; before the microscope and other instruments of precision had elucidated the physiology and pathology of the blood and blood-forming organs. In those days the pallor of the skin and mucous membranes was attributed to "a diminution of the amount of blood," and even at the present day there are physicians who still share in this fallacy. From a pathological standpoint, the chief features of the so-called anemic state, are the morphological and chemical changes in the blood,—its qualitative deterioration. It, therefore, has been suggested that the term dysemia (bad blood) would more closely describe the conditions present. However, a word which has the sanction of usage for so many years cannot be easily displaced, and will probably long survive in medical nomenclature.

There is only one criterion on which to base a diagnosis of dysemia, and that is the changes in the blood. The other symptoms to which much significance was formerly attributed,—pallor of the skin and mucous membranes, rapid pulse, and palpitation, general weakness, vertigo, and the like,—are of doubtful value, since they are present in various conditions in which the state of the blood is normal. Thus, for instance, they may result from purely psychical causes,—fear or anxiety,—or occur in the course of chronic digestive disorders, or neurasthenia.

To estimate the degree of dysemia the diminution in the percentage of hemoglobin is of the greatest importance, and this may range within wide limits, even to one-fifth of the normal proportion. Next in importance is the reduction in the number of red blood corpuscles, which, however, is perceptible only in the more marked cases. In the severe types of the disease reductions to 50 per cent. are quite frequent. There is also a change in size and shape of the red blood-cells, known collectively as poikilocytosis, in the pronounced form of dysemia. Usually the corpuscles are diminished in size and their shape becomes irregular; there may be granular deposits in their protoplasm. Sometimes there may be found in the blood the so-called normoblasts, which are nucleated red cells, resembling those present in the red bone marrow. The latter have been specially observed in dysemia, following profuse losses of blood, but in any case are of no significance.

These remarks apply only to the simple types of dysemia and not to the progressive pernicious forms, characterised by changes in the blood-forming tissues. As pointed out by Ehrlich and Lazarus in an excellent article on this subject, to which I am indebted for much information, the radical difference between the simple and pernicious varieties of dysemia is that in the former the regeneration of blood takes place in a physiological manner, while in the latter it reverts to the embryonic type, as shown by the presence of foreign elements. The majority of cases of simple dysemia are secondary to diseases attended with malassimilation, tissue waste, hemorrhages, and profuse discharges, while it is important to remember that it is often due to a condition of autotoxemia, or to the absorption of toxines generated by bacteria. It is, therefore, not enough to make a diagnosis of dysemia, but the primary cause should be sought and removed if possible.

The treatment of the changed condition of the blood consists in the adoption of an appropriate regimen,—a nutritious dietary, fresh air, and sunshine,—in connection with the administration of iron supplemented occasionally with arsenic. Hydrotherapy is a very valuable auxiliary in some cases. The patient should rest as much as possible, and in severe cases should take a vacation in the mountains. Long before modern hematology had its beginning, iron was administered on empirical grounds, and all that modern medicine has contributed to the therapy of dysemia is the introduction of ferruginous compounds, which are not only superior in efficacy to those in former use, but free from their objectionable features. The chief disadvantages of the older iron preparations were their disturbing effect upon the digestion, their tendency to produce constipation, and their destructive action upon the teeth. It is a notable achievement of pharmaceutical chemistry to place at the physician's disposal organic ferruginous compounds, which approximate closely in composition to the form in which iron is contained in the red blood-globules. The most prominent preparation of this kind is peptomangan (Gude). This consists of iron and manganese in the form of peptonates, which, representing albuminous elements in their last stage of digestion, are immediately absorbed and assimilated, without undergoing any previous transformation in the gastrointestinal tract. The presence of manganese in combination with iron in peptomangan is based upon the fact that both of these elements are found associated in the red globules.

Having had my attention directed to this preparation through the reports of leading authorities in European and American journals, I subjected it to a thorough test in the Hotel Dieu Hospital, Montreal, and have briefly recorded here the histories of a number of typical cases in order to demonstrate its efficiency in dysemia, as shown by the rapid increase of the hemoglobin percentage and number of red blood cells.

Case I.—A woman, aged 25 years; dysemia. Time of administration, 30 days. First count, 3,376,400 red corpuscles to the c.mm.; second count, 4,400,300 to the c.mm. Hemoglobin: first examination, 51 per cent.; second examination, 70 per cent.

Case II.—A girl, aged 20 years; dysemia. Time of administration, 30 days. First count, 2,630,200 red corpuscles to the c.mm.; second count, 3,970,000 to the c.mm. Hemoglobin: first examination, 40 per cent.; second examination, 60 per cent.

Case III.—A man, aged 25 years; dysemia, following typhoid fever. Time of administration, 30 days. First count, 2,500,200 red corpuscles to the c.mm.; second count, 3,950,000 to the c.mm. Hemoglobin: first examination, 39 per cent.; second examination, 50 per cent.

Case IV.—A woman, aged 39 years; dysemia. Time of administration, 30 days. First count, 2,750,400 red corpuscles to the c.mm.; second count, 3,500,000 to the c.mm. Hemoglobin: first examination, 35 per cent.; second examination, 60 per cent.

Case V.—A woman, aged 35 years; dysemia, following miscarriage. Time of administration, 30 days. First count, 2,800,000 red corpuscles to the c.mm.; second count, 3,300,000 to the c.mm. Hemoglobin: first examination, 33 per cent.; second examination, 45 per cent.

Case VI.—A young girl, aged 17 years; dysemia, following typhoid fever. Time of administration, three weeks. First count, 2,495,270 red corpuscles to the c.mm.; second count, 3,300,200 to the c.mm. Hemoglobin (percentage of normal amount): first examination, 35 per cent.; second examination, 45 per cent.

Case VII.—A young boy, aged 16 years; dysemia, following typhoid fever. Time of administration, three weeks. First count, 3,670,000 red corpuscles to the c.mm.; second count, 4,600,300 to the c.mm. Hemoglobin (percentage of normal amount): first examination, 40 per cent.; second examination, 65 per cent.

Case VIII.—A man, aged 30 years; dysemia, following amputation of the leg. Time of administration, three weeks. First count, 2,360,400 red corpuscles to the c.mm.; second count, 3,500,200 to the c.mm. Hemoglobin (percentage of normal amount): first examination, 30 per cent.; second examination, 70 per cent.

Case IX.—Woman, aged 24 years; dysemia, following pneumonia. Time of administration, three weeks. First count, 2,600,250 red corpuscles to the c.mm.; second count, 3,400,000 to the c.mm. Hemoglobin (percentage of normal amount): first examination, 35 per cent.; second examination, 70 per cent.

Case X.—Woman, aged 20 years; dysemia, following miscarriage. Time of administration, three weeks. First count, 2,502,600 red corpuscles to the c.mm.; second count, 4,006,200 to the c.mm. Hemoglobin (percentage of normal amount): first examination, 40 per cent.; second examination, 65 per cent.

Case XI.—Man, aged 32 years; anemia, following typhoid fever. Time of administration, three weeks. First count, 2,300,-

000 red corpuscles to the c.mm.; second count 3,640,160 to the c.mm. Hemoglobin (percentage of normal amount): first examination, 33 per cent.; second examination, 62 per cent.

Case XII.—A girl, aged 16 years; dysemia. Time of administration, four weeks. First count, 2,290,700 red corpuscles to the c.mm.; second count, 3,800,200 to the c.mm. Hemoglobin (percentage of normal amount): first examination, 40 per cent.; second examination, 60 per cent.

Case XIII.—A girl, aged 16 years; dysemia. Time of administration, four weeks. First count, 2,430,300 red corpuscles to the c.mm.; second count, 4,000,300 to the c.mm. Hemoglobin (percentage of normal amount): first examination, 40 per cent.; second examination, 65 per cent.

Comparing my results with peptomangan (Gude), with those obtained from other chalybeates of this class, I have been led to give it decided preference. As already stated, the only reliable means of diagnosticating dysemia is by the examination of the blood, and for the same reason the only way of testing the efficiency of a ferruginous preparation is by making blood-counts and estimating the percentage of hemoglobin. On the ground of my findings, as shown by the histories of the cases cited, the results of such tests have been uniformly satisfactory and entitle the preparation to a leading place in ferruginous medication.

